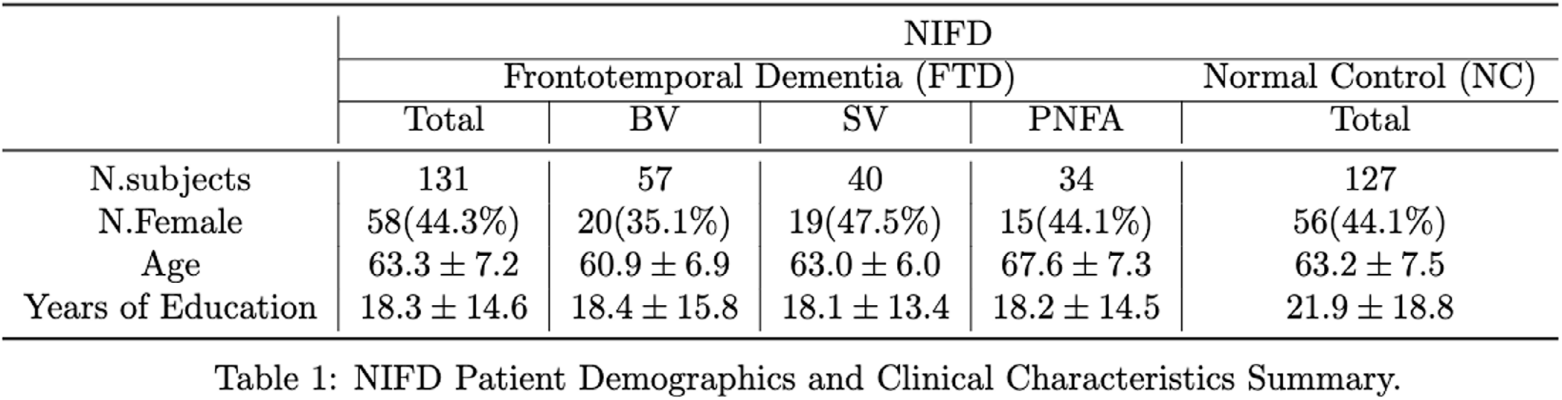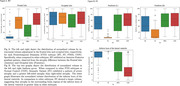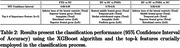# Automated Brain Volumetry Analysis for Differential Diagnosis of Frontotemporal Dementia Subtypes

**DOI:** 10.1002/alz.086728

**Published:** 2025-01-09

**Authors:** Hyeonwoo Cho, Mina Park, Seung Hyun Lee, Wooseok Jung, Dong‐Hee Kim, Jinyoung Kim, Yeha Lee, for the Frontotemporal Lobar Degeneration Neuroimaging Initiative*

**Affiliations:** ^1^ VUNO Inc., Seoul Korea, Republic of (South); ^2^ Gangnam Severance Hospital, Yonsei University College of Medicine, Seoul Korea, Republic of (South); ^3^ VUNO Inc., Seocho‐gu, Seoul Korea, Republic of (South)

## Abstract

**Background:**

Clinical diagnosis of frontotemporal dementia (FTD) can be challenging, requiring an accurate tool dedicated to this diagnostic hurdle. Since FTD exhibits distinct regional atrophy patterns on magnetic resonance imaging (MRI), AI‐aided automated brain volume analysis could enhance the clinical diagnostic assessment of FTD, including the detection of the disease and the classification of subtypes, which encompass behavioral variant (BV), semantic variant (SV), and progressive non‐fluent aphasia (PNFA). In this study, we leverage automated brain volumetry software to approach both FTD detection and the differential diagnosis among its subtypes.

**Method:**

A total 258 patients with FTD (BV = 57, SV = 40, PNFA = 34) and 127 Normal Control (NC) were identified from the frontotemporal lobar degeneration neuroimaging initiative (NIFD) data sets with available MRI. We obtained 104 areas of patient’s normalized regional brain volumes using VUNO‐Med DeepBrain. Comparative analyses were conducted to explore differences in regional volume distribution between FTD and NC, as well as among FTD subtype based on their known atrophy patterns. Additionally, regional normative percentiles were utilized as features for classification using the XGBoost algorithm to distinguish FTD vs NC, BV vs SV vs PNFA, and BV vs SV vs PNFA vs NC.

**Result:**

In BV, significant differences in atrophy patterns were observed in the frontal lobe, demonstrating an anterior‐posterior gradient compared to SV, PNFA and NC (P<0.01). SV exhibited left side dominant asymmetric atrophy, particularly in the inferior temporal lobe, compared to BV, PNFA, and NC (P<0.01). Additionally, the inferior horn of the lateral ventricle in SV had higher volume than other subtypes, attributable to atrophy of surrounding brain regions. In contrast, the differences in atrophy patterns for PNFA were statistically less significant when compared to other FTD subtypes. Automated brain volumetry software‐based classifications demonstrated high accuracy of FTD vs NC (Accuracy 95% CI = [0.88, 0.89]), BV vs SV vs PNFA ([0.79, 0.81]), and BV vs SV vs PNFA vs NC ([0.82, 0.83]).

**Conclusion:**

Our study demonstrates the effectiveness of automated brain volumetry and machine learning in identifying FTD and differentiating frontotemporal dementia subtypes, contributing to an enhanced understanding of subtypes and improved diagnostic precision in neurodegenerative disorders.